# Bilateral Inverted and Impacted Mandibular Third Molars: A Rare Case Report

**DOI:** 10.7759/cureus.36573

**Published:** 2023-03-23

**Authors:** Yara Mohamed Talib, Nouf Waleed Albalushi, Doha Mohamed Fouad, Ahmed M Salloum, Bhavna Jha Kukreja, Hossam Abdelmagyd

**Affiliations:** 1 Preventive Dental Sciences, Thumbay Dental Hospital, College of Dentistry, Gulf Medical University, Ajman, ARE; 2 Oral and Maxillofacial Surgery/Diagnostic and Surgical Dental Sciences, Thumbay Dental Hospital, College of Dentistry, Gulf Medical University, Ajman, ARE; 3 Periodontics/Preventive Dental Sciences, Thumbay Dental Hospital, College of Dentistry, Gulf Medical University, Ajman, ARE

**Keywords:** case report, mandible, third molar, impaction, inverted

## Abstract

Even while certain forms of mandibular impactions (such as inverted molars) might be considered unusual findings, mandibular impacted teeth are really one of the most regularly seen dental abnormalities. Two female patients’ mandibular third molars were discovered to be inverted during a regular inspection, and two such examples are reported here in this article. Both patients underwent routine radiographic examination. Cone beam computed tomography and orthopantomogram were requested to evaluate the state of the bone and to check for any abnormalities, and inverted impacted teeth were discovered. A tooth is said to be inversed when it is placed reversed and seated upside down. Ascending ramus is the most common site for third molars in the mandible. It is also possible for a maxillary tooth to get impacted and for the tooth to be pushed all the way to the orbit's floor, though mandibular impacted teeth are more common. Only a few cases of inverted and impacted mandibular third molars have been reported in the literature. No definitive treatment protocols exist for the removal of inverted teeth. The safest protocol is conservative treatment in which the teeth are not extracted until they produce pathological signs.

## Introduction

When a tooth is unable to erupt normally because of a lack of room or a blockage in the eruptive route, we say that the tooth is impacted. As determined by the degree of the angle between the second and third molars' long axis. The crown of the impacted third molar is most important as it is the starting point for this measurement [1]. When the long axis of the impacted third molar and second molars are parallel, then the molar is said to be vertically impacted. When the long axis of the second molar and impacted molars are converging coronally, then the impacted molar is mesioangular. In the distoangular impacted third molar, the long axis of the second molar and impacted third molars converges apically. In horizontal impaction, the long axis of the second molar and that of the impacted third molar are perpendicular to each other. The position of the tooth is buccolingual in a person with inverted impaction; this is called buccolingual impaction.

Using the Pell and Gregory classification as a reference, one may decide on an appropriate impaction level [2]. In position A, the impacted tooth's occlusal level is parallel to that of the surrounding teeth. When the impacted tooth's occlusal level is midway between the occlusal plane and cervical line of the adjacent tooth, this is known as position B. The impacted tooth's occlusal level is apical to the cervical line of the adjacent tooth in position C. When the impacted third molar's crown does not extend into the ramus and the molar is visible in front of the ramus's anterior border, this is known as position I. When the crown of the tooth is outside of the mandibular ramus, this is position II. More than half of the crown must be contained within the ramus to classify as position III [2].

American Dental Association (ADA) and the American Association of Oral and Maxillofacial Surgeons (AAOMS) classify the impacted tooth as ADA codes. Although maxillary and mandibular molars are the most often affected teeth, only in a small number of instances inverted impaction of teeth is documented. When it comes to the mandible, the ascending part of the ramus of the mandible is the usual site for the inverted wisdom tooth. Cases have been reported where the impacted molar in the maxilla is displaced up to the level of floor of the orbit [3].

In this case report, two cases of bilateral impacted mandibular molar are reported which are inverted. For the radiographs and scans to be published, patients gave their written informed consent.

## Case presentation

Using the Pell and Gregory Classification based on the occlusal level of the tooth, in position A, the impacted tooth's occlusal level is parallel to that of the surrounding teeth. When the impacted tooth's occlusal level is midway between the occlusal plane and the cervical line of the adjacent tooth, this is known as position B. The impacted tooth's occlusal level is apical to the cervical line of the adjacent tooth in position C. When the impacted third molar's crown does not extend into the ramus and the molar is visible in front of the ramus's anterior border, this is known as position I. When the crown of the tooth is outside of the mandibular ramus, this is position II. More than half of the crown must be contained within the ramus to classify as position III.

ADA and AAOMS classify the impacted tooth as 07220 wherein overlying smooth tissue (impaction that calls for incision of overlying smooth tissue and the elimination of the teeth), 07230 which incorporates partly bony impacted tooth (impaction that calls for incision of overlying smooth tissue, elevation of a flap, and both elimination of bone and teeth or sectioning and elimination of teeth), 07240 which suggests absolutely bony impaction (impaction that calls for incision of overlying smooth tissue, the elevation of a flap, elimination of bone, and sectioning of teeth for elimination), and 07241 absolutely bony, with uncommon complications (impaction that calls for incision of overlying smooth tissue, the elevation of a flap, elimination of bone, sectioning of the teeth for elimination, and/or presents uncommon problems and circumstances).

Case 1

A female patient, age 50, from Jordan came into the dentist's office complaining of persistent bleeding while brushing and missing front teeth in the mandible and pain in the lower jaw region while chewing food which was occasional. Upon clinical evaluation, it was established that the patient has poor oral hygiene with an inordinate amount of plaque and calculus deposition causing inflammation and recession of the gingiva. Calling for routine radiographic examination, an orthopantomogram (OPG) was requested to evaluate the state of the bone and to check for any abnormalities. As a result, bilateral, impacted third molars were discovered which were inverted.

**Figure 1:**
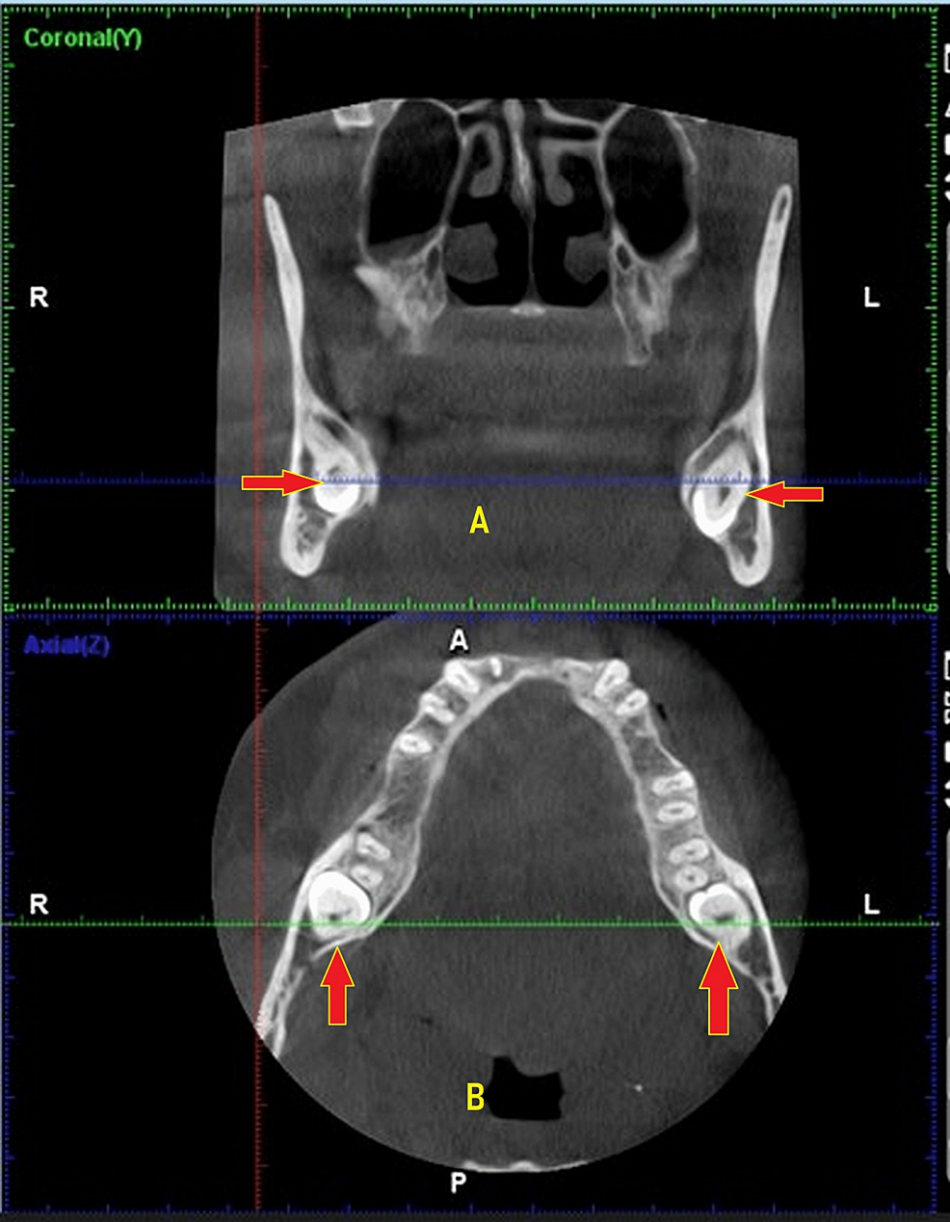
Coronal and axial CBCT views of the mandible showing bilateral inverted impacted mandibular third molars A: Coronal-section CBCT B: Axial-section CBCT CBCT: Cone beam computed tomography

**Figure 2:**
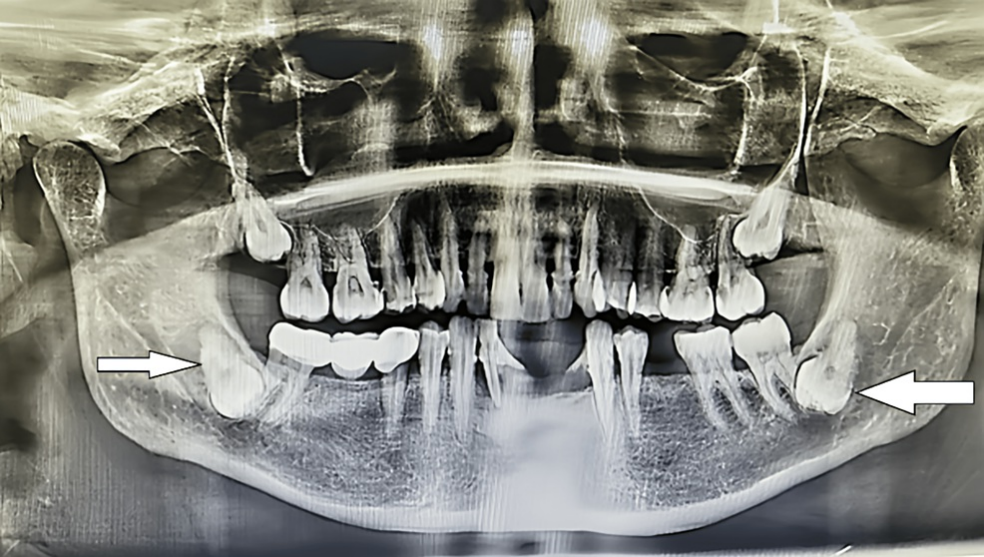
Orthopantomograph showing bilateral Class III, Position C, ADA-AAOMS 07241, inverted impacted mandibular third molars

**Figure 3:**
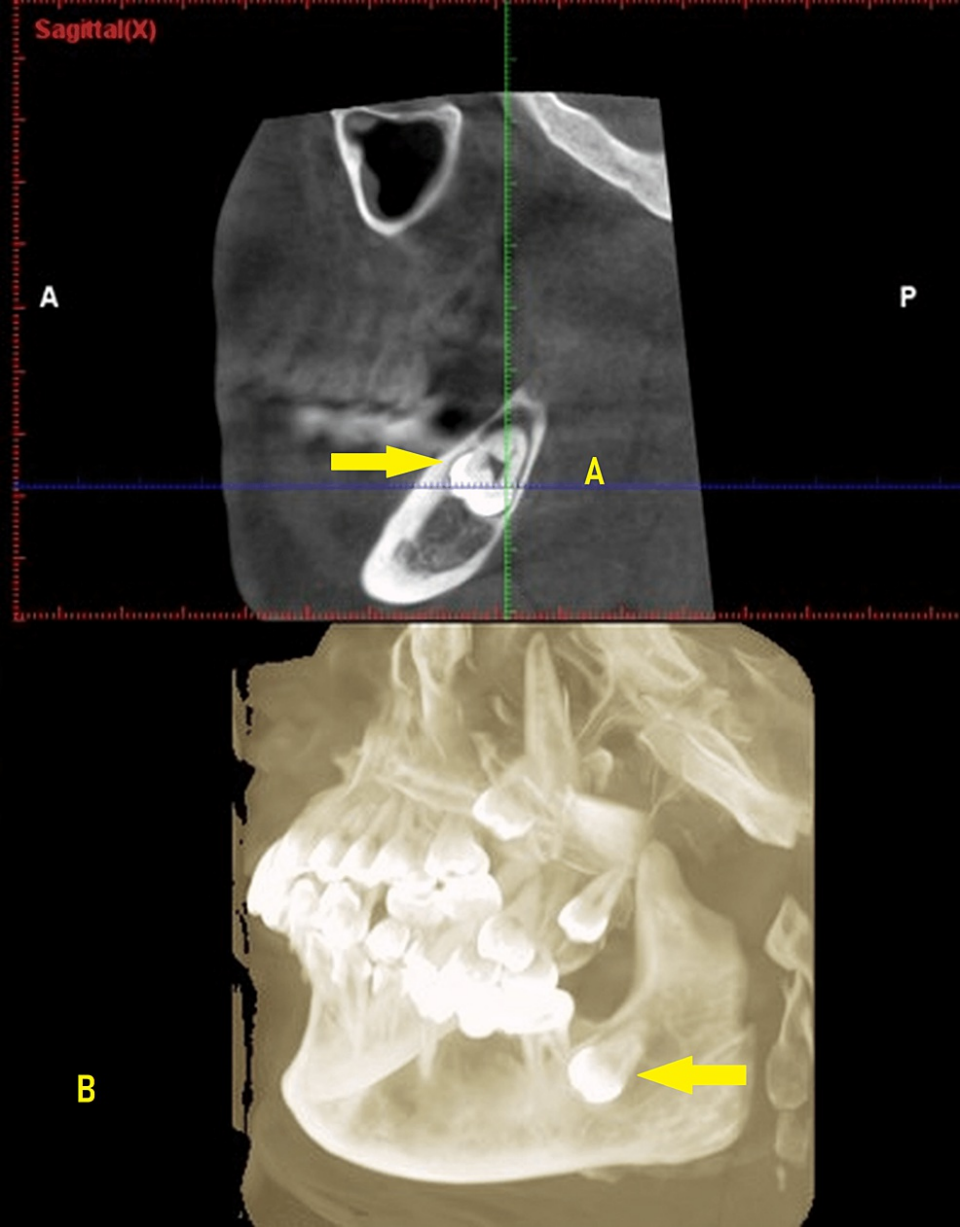
Sagittal-view CBCT and lateral oblique (right side) views of the mandible showing Class III position C inverted impacted mandibular ADA-AAOMS 07241 third molar A: Sagittal section CBCT B: Three-dimensional reconstruction of sagittal section CBCT: Cone beam computed tomography

As shown in Figure 1, coronal and axial cone beam computed tomography (CBCT) views of the mandible showed bilateral inverted impacted mandibular third molars. Impacted 18 and 28 were radiographically visible in the OPG (Figure 2). Sagittal-view CBCT and lateral oblique (right side) views of the mandible are the evidence of inverted impacted mandibular third molar (Figure 3).

 

 

The impacted molar's crown was pointing down toward the mandibular apex. The patient was advised to get her impacted teeth removed, but she refused to undergo the surgery as she was more concerned for the treatment of her other dental problems. Therefore, it was recommended that the patient come in for regular follow-up visits.

Case 2

**Figure 4:**
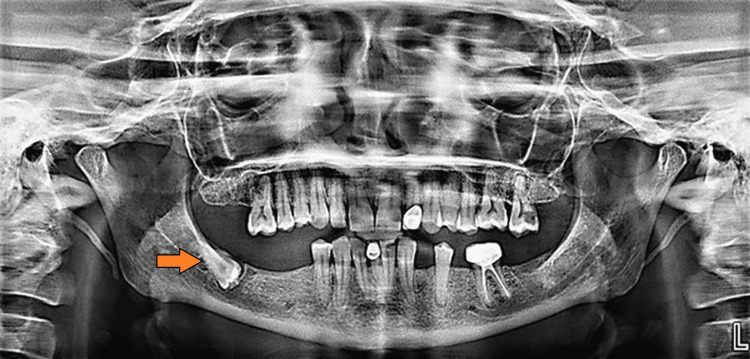
Orthopantomograph showing inverted impacted mandibular third molar ADA-AAOMS 07241 on the right side

A 32-year-old female patient from Syria reported to the dental clinic with a chief complaint of having severe pain in relation to 27 with periapical infection and distal caries. Upon clinical evaluation, it was established that the patient has poor oral hygiene with an inordinate amount of plaque and calculus deposition and gave history of loss of teeth due to decay. An OPG was requested for routine bone evaluation (Figure 4). An impacted and inverted third molar was observed in the OPG.

## Discussion

Tooth germs of the mandibular molar normally form by nine years of age. Cusp mineralization takes place two years further. In the eleventh year, teeth are placed in the anterior border of the ramus, with the occlusal floor going through anteriorly. Crown formation is finished by 14 years, and fifty percentage of the root is shaped by the age of sixteen years. Root forms with open apex by 18 years, and by 24 years, 95% of entire third molars normally erupt. An impacted tooth is a tooth that is absolutely or in part unerupted and is located in opposition to any other teeth, bone, or tender tissue so that its eruption is unlikely, defined in keeping with its anatomic function. A malposed tooth is a tooth, unerupted/erupted that is in a bizarre function within the maxilla/mandible. An unerupted tooth is a tooth that has no longer perforated the oral mucosa e.g., a tooth previous to emergence, or a tooth not able to erupt out or emerge from the dental alveolar tissues into the oral cavity. A partially erupted tooth is a tooth that has not erupted right completely, however, has crossed the bone barrier and has no longer reached the occlusal line into a regular useful functional position.

Inversion is a malposition of a tooth where it has reversed in the upside-down position [2]. Inverted impacted teeth might also present within the jaws for a long term with non-specific manifestations. However, they may result in headache, crowding, diastema, late or ectopic eruption, expulsion of teeth into the floor of the nasal cavity, loss of adjacent teeth, and worsening of existing conditions [4]. Incisors, canines, and premolars have all been reported to have inverted impactions [5-7]. Molars were seldom noted as a source of discomfort or impaction. Tooth's inversion is due to a peculiar growth of odontogenic epithelium before the formation of the teeth bud [2]. A tooth in the maxilla may be knocked out anywhere between the gums and the orbital floor, but the most typical site of injury to the third molar in the mandible is the ejecting ramus [3]. Because of the relatively peculiar position of the crown and the root, this kind of impaction has been labeled as complex.

Tooth impaction can be caused by a number of underlying systemic conditions, including but not limited to genetics, endocrinology, radiation, cleidocranial dysplasia, febrile illness, Down syndrome, and other factors such as an abnormal eruptive pathway, supernumerary teeth, malposed teeth microorganisms, retention of deciduous teeth, deficiency of arch length, odontogenic tumors, and cleft lip/palates [8]. When it comes to removing impacted teeth from the sinus and infratemporal fossa during surgical procedures, radiographic imaging plays a significant role in determining the precise location of inverted, impacted teeth and their interaction with critical anatomic structures. Moreover, this enables their elimination with minimum surgical trauma [9].

Mesioangular is one of the types of impaction that is the most common type of mandibular molar impaction [5]. Of the 1200 subjects whose pantographic radiographs were examined by Padhye et al., 33.33 percent showed mesial side alignment of molars [9]. Kumar et al. found that 52.89 percent of Eritrean citizens were having mesioangular impacted molars [10]. In 2016, studies showed the prevalence of mesial-perspective impaction in 47.1% of patients, using data provided by Nagaraj and co-authors in addition to many different researchers [11-16]. According to studies by Al-Dajani et al. and Yilmaz et al., vertical impaction is more frequent [17,18]. One study found that 40.7% of patients had this impaction, whereas only 7.1% had mesioangular impaction. Another study found that 53.0% of patients had vertical impaction and 29.0% had mesioangular impaction. These findings were contradictory to the findings of Al-Dajani et al. and Yilmaz et al. [17,18]. The impacted molar's angle was determined by the researchers using the second and third molar's long axes. Using the second and third molars' long axes, the researchers calculated the obtuseness of the impacted tooth. Within the measurements themselves, the axis of the second tooth became the new reference point. It became possible to gauge the degree to which the tooth axis deviates from the opposing axis. When the angle between the teeth and the reference line is less than 10 degrees on each side, it is considered to be vertically impacted. This change seems to be equal to angles used by Tetsch and Wagner's classification [19]; nevertheless, Al-Dajani and Yilmaz measured the amplitude from the axis of the second one tooth, opposite to the Tetsch and Wagner classification, which measures the perspective among the occlusal plane and the axis of the impacted teeth. Winter’s unique classification, with the aid of using design, does now no longer deliver a cost for the perspective of teeth deviation and the opportunity of deviation from the lengthy axis of the second one tooth, however most effective the spatial dating of the lengthy axes [20].

Experts from Hong Kong cited horizontal impaction as the most common kind of impaction [19]. In the sample size of 7486 people, they found 47.45% were having horizontal impaction, with 42.45% of those people having an impacted tooth. The distoangular type was the second most prevalent form of impaction, making up 39.04% of all impactions. This finding was in confirmation of the findings of Winter GB, who also described a chronological sequence of events for each kind of impaction [20]. In 700 studied subjects, 63.99% of the impacted mandibular molars were mesial-angular, whereas 20.56 % were distoangular. Al-Anqudi et al. further offered a comparable sample of character collision occurrences [13]. The foremost second maximum not unusual place impaction withinside the literature is the vertical function [21,22]. It is possible that a little departure of the crown of the impacted teeth from the long axis of the second molar was misinterpreted as upward arrangement by the authors of the various courses, leading to a large mistake in data on the occurrence of vertical and distal-point impaction. While there was no plausible deviation from a parallel long axis in the mandibular second molars, this fact did not prevent impaction from being labeled as vertical. Authors in the required reading for this course agree that our case study's rearranged or unexpected impaction is rather extraordinary [19,21,22].

The National Institute for Clinical Excellence (NICE) guidelines for the management of third molars advise against routine preventive removal of pathology-free teeth third molars with impacts. The NICE guidelines state that only people with pathology, such as unrestorable caries, untreatable pulpal and/or peri-apical pathology, cellulitis, abscess, osteomyelitis, internal or external resorption of the tooth or adjacent teeth, recurrent episodes of pericoronitis, fracture of the tooth, disease of the follicle including cyst/tumor, tooth/teeth impending surgery, or reconstructive dentistry. Third molars that are partially or completely impacted by soft tissue are far more likely to develop plaque buildup and pericoronitis [23]. Due to the age of the person and deeper positioning of the inverted tooth, its extraction is more complex than the extraction of a normally impacted tooth. The teeth are firmly set inside the bone, therefore significant amounts of bone must be removed during surgery. Several methods, including the use of rotary burs, chisel mallets, Lasers, piezosurgery, etc., may help achieve this goal. While removing maxillary impacted third molars, an oro-antral fistula may form, or a piece of tough tissue may dislodge in the sinus, nose, or infratemporal fossa [23,24]. One of the most important essential stages in removing the inverted tooth is the osteotomy procedure; however, there are a variety of possible approaches, and they may be harmful if employed by an inexperienced practitioner. Rotating cutting machines, on the other hand, are undeniably harmful since the excessive heat they produce, generally while cutting the bone, may lead to moderate osteonecrosis and slow down the healing process [25]. Furthermore, the treatment protocol for such cases is not available in the literature, clinicians need to weigh the costs and advantages of treating impacted molars immediately. Prior to any surgical removal, the risks involved should be fully explained to the patient, and they should sign a written consent form acknowledging that they understand the potential consequences of the surgery [26].

In 1988, an Italian oral surgeon named, Vercellotti developed piezosurgery, in an effort to overcome the limitations of conventional equipment in oral bone surgical technique, by employing augmenting and improving traditional ultrasonic technology [27]. Piezosurgery is a kind of osteotomy in which ultrasonic micro-vibrations are used to perform green bone slicing [28]. Ultrasonic vibrations grant a specific and depicted slicing activity, leading to a higher level of accuracy and protection and substantially less tissue injury than the utilization of typically used burs [29-32]. This makes the piezoelectric device useful for application in critical areas, including the posterior mandible, where the osteotomy is required in close proximity of nerves and blood vessels.

For the removal of teeth that are inverted, there are no established therapeutic guidelines. The safest course of action is conservative treatment, which postpones tooth extraction until pathological symptoms appear. To find these changes as soon as feasible, the patient should undergo routine clinical and radiological evaluations. Also, the patient should be included in the management decision and informed of the indications, contraindications, risks, and advantages of conservative care and surgical removal of impacted teeth. As mentioned in the present case report, if it is an accidental finding, then the prevention of further progression of any disease can be done.

The limitation of the present case report is that as it is not a rare finding, a larger sample study and more cases should have been gathered.

## Conclusions

Conservative treatment is the treatment of choice for the bilateral inverted lower third molars because if they are pathology-free and fully covered by bone and mucosa, which constitute effective barriers against bacterial invasion. Additionally, the medical condition and age of the patient as well as the anticipated local complications associated with removal of the teeth must also be considered. Surgical extraction of inverted third molars with inapproachable positions requires an aggressive bone removal on bilaterally, which is a major disadvantage.
